# Physiotherapy‐Led Rehabilitation of Foot Drop Following Lower Limb Gunshot Injury

**DOI:** 10.1002/ccr3.71930

**Published:** 2026-01-24

**Authors:** Mahmuda Akter Akhi, Kazi Md Azman Hossain, Sabrina Tina, Md. Abdul Alim

**Affiliations:** ^1^ Department of Physiotherapy Bangladesh Health Professions Institute (BHPI) Dhaka Bangladesh; ^2^ Department of Physiotherapy and Rehabilitation Jashore University of Science and Technology (JUST) Jashore Bangladesh; ^3^ Department of Physiotherapy Centre for the Rehabilitation of the Paralysed (CRP) Dhaka Bangladesh

**Keywords:** foot drop, gunshot injury, lower limb, physiotherapy, rehabilitation

## Abstract

Gunshot injury (GSI) severely damages muscles and the nervous system, requiring long‐term treatment and rehabilitation. This case report highlights the effectiveness of physiotherapy‐led rehabilitation in a patient with lower limb injury and foot drop. After emergency treatment, the patient was diagnosed with common peroneal nerve injury (CPNI) and referred to a rehabilitation center, where evaluation revealed foot drop, discomfort, paresthesia, decreased mobility, and functional limitations. A structured rehabilitation program was then initiated that included education, orthotic devices, stretching, range of motion exercises, strength training, electrical stimulation, sensorimotor exercises, and mobility training. After treatment, the patient's pain, muscle strength, range of motion, functional capacity, and quality of life improved significantly, freeing him from wheelchair dependence and regaining the ability to stand and walk independently. This case demonstrates that timely physiotherapy‐led rehabilitation is crucial for the recovery and restoration of independence in a post‐GSI with foot drop patient.

## Key Clinical Message

Primary physiotherapy rehabilitation can resolve dysfunction, accelerate nerve regeneration, and improve quality of life in foot drop caused by common peroneal nerve injury after lower limb gunshot injury in a resource‐limited setting.

## 1 Introduction

Gunshot injury (GSI) is a significant global health problem, often causing complex damage to muscles, bones, and nerves. Peripheral nerve injury (PNI) is particularly prominent, accounting for approximately 25%–36% of GSI cases [1]. Nonfatal gunshot injury occurs approximately five times more frequently than fatal injuries [2], increasing the need for comprehensive and integrated rehabilitation services. The common peroneal nerve (CPN) is the most commonly affected nerve in lower limb injuries. This often results in foot drop, which causes difficulty in dorsiflexion and difficulty in walking. This condition severely affects the patient's mobility and independence. Nerve damage mechanisms include direct trauma from the bullet and secondary effects like cavitation and shockwaves, resulting in neuropraxia, axonotmesis, or neurotmesis. Clinically, these injuries manifest as motor deficits, sensory disturbances, and neuropathic pain. Recovery can be obstructed by complications such as nerve enlargement, retraction, and scar tissue if early intervention is lacking [3, 4, 5, 6, 7]. During a national antidiscrimination movement last year, many individuals were injured during clashes. Explosives, tear gas, and rubber bullets caused numerous injuries, especially among students and young protesters [8, 9]. Many lower‐limb GSIs, especially in low‐income communities, went untreated or poorly rehabilitated. In cases of CPNI and foot drop, early physiotherapy‐based rehab is crucial for optimal recovery. A well‐structured physiotherapy‐led rehabilitation program, which includes comprehensive range of motion exercises, muscle strengthening, and gait training, can help prevent muscle weakness and joint contractures and promote nerve recovery. Research has shown that early initiation of physiotherapy can improve motor skills and sensory abilities, helping individuals to regain their function more effectively [10, 11, 12, 13]. This case report details the rehabilitation of a young man with foot drop due to CPNI in GSI, further highlighting the importance of a comprehensive physiotherapy and rehabilitation approach in restoring function and independence.

## 2 Case Presentation

**FIGURE 1:**
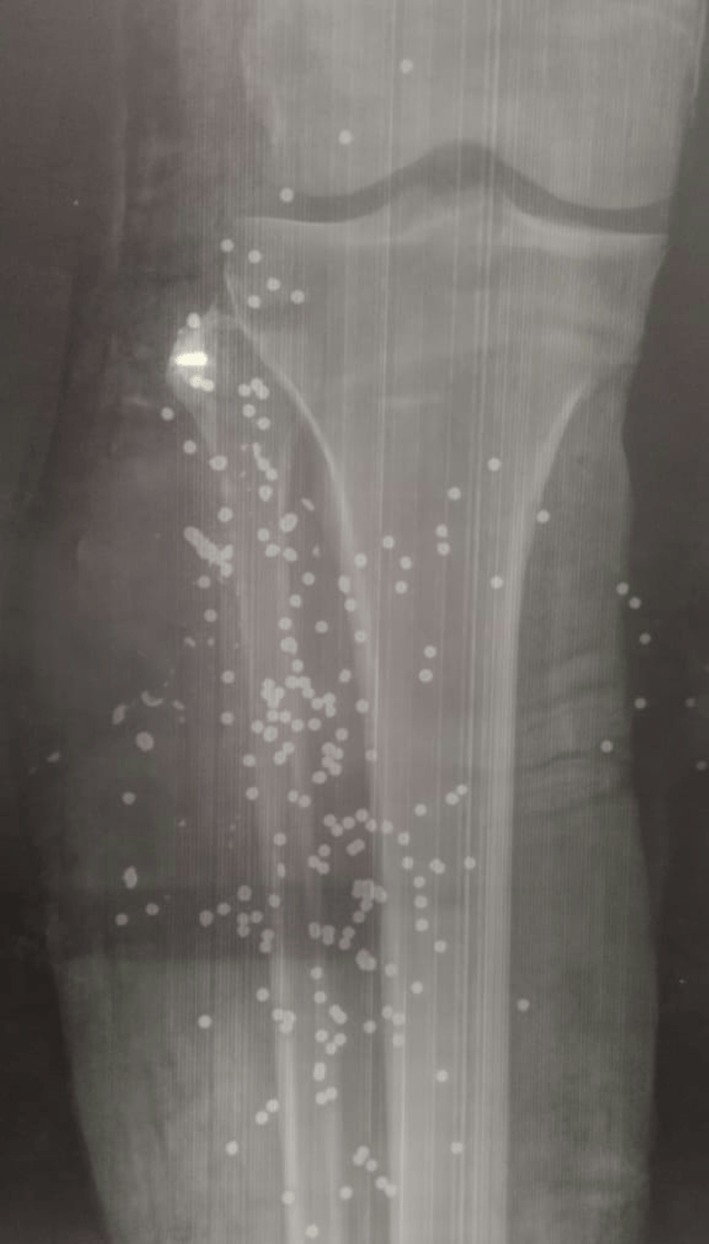
X‐ray image displaying bullet fragments.

**FIGURE 2:**
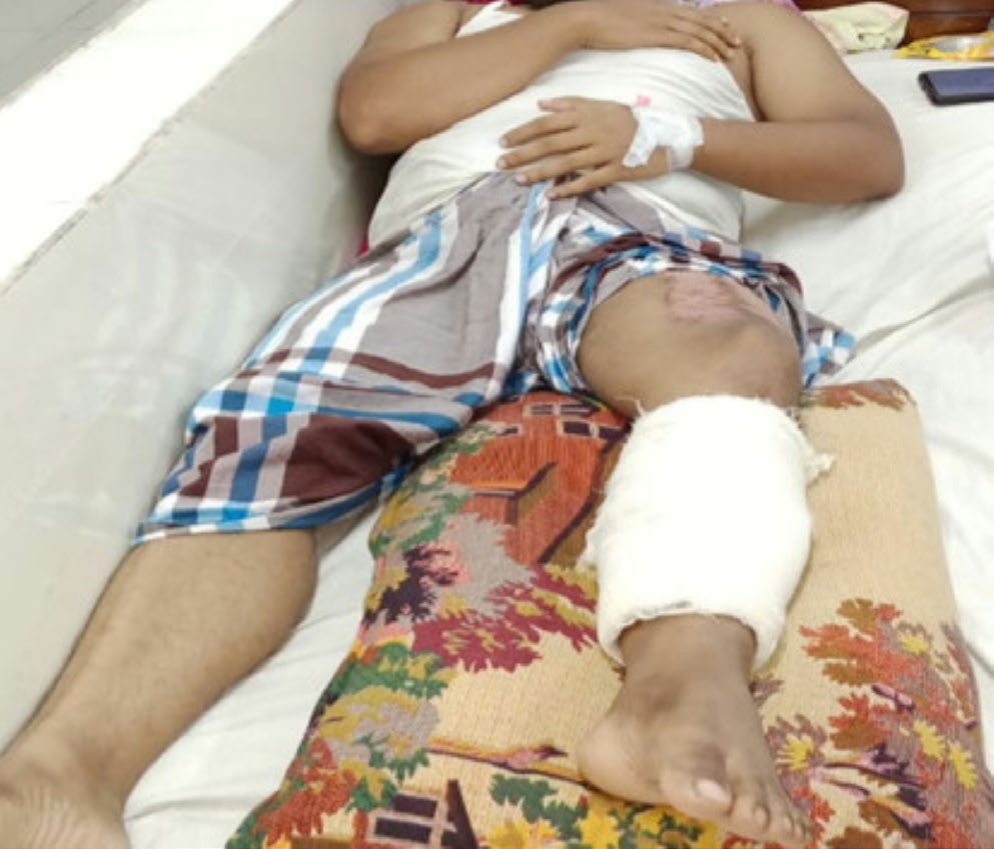
Patient with a gunshot injury receiving initial medical assistance.

During the nationwide antidiscrimination movement, a 32‐year‐old young man was brought to a local hospital after suffering multiple GSI to his left lower leg. He didn't have any significant medical history, such as diabetes, high blood pressure, heart disease, or kidney problems. Plus, he had no history of alcohol use or smoking. Following the gunshot injury, the patient received staged surgical and medical management to stabilize the wound, control infection, and preserve viable soft tissue. Initial surgical intervention included thorough wound exploration, debridement of devitalized tissue, and removal of retained bullet fragments to reduce contamination and support tissue healing. During the early postoperative period, signs of wound infection and soft‐tissue compromise necessitated additional surgical procedures, including repeated wound care and split‐thickness skin grafting to reconstruct the affected area and promote durable wound closure. In total, the patient underwent three surgical procedures, all focused on soft‐tissue management rather than direct nerve repair. No immediate nerve exploration or reconstructive nerve surgery was performed because there was no evidence of vascular injury, complete nerve transection, or limb‐threatening complications, and the neurological deficits were considered potentially recoverable with conservative management. Postoperatively, the patient received intravenous broad‐spectrum antibiotics during the acute phase, followed by oral antibiotics after clinical improvement, to control infection and support wound healing. Analgesics and supportive medications were also administered as needed for pain control and comfort. Once the wound condition stabilized and infection was adequately controlled, the patient was referred for comprehensive physiotherapy‐led rehabilitation to address functional impairments related to the peripheral nerve injury (PNI). Post‐traumatic PNI with foot drop was diagnosed due to gunshot trauma. A structured physiotherapy and rehabilitation program was developed to support his recovery, enhance his mobility, and assist him in reintegrating into the community. He actively volunteered to participate in the program (Figures 1 and 2).

## 3 Investigation

**FIGURE 3:**
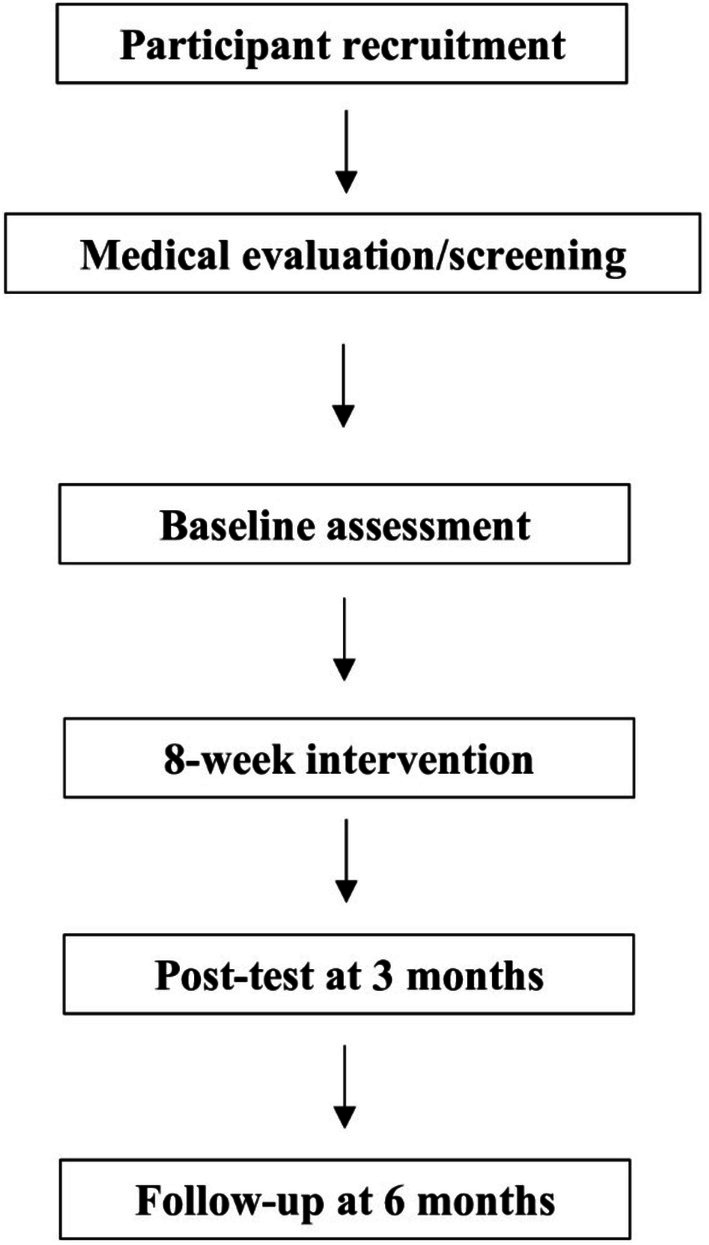
Study flow diagram.

Upon arrival at the Rehabilitation Center, the patient was seated in a wheelchair and exhibited clinical signs indicative of left‐sided foot drop. The physiotherapy team performed a thorough physical and neurological assessment. The neurological examination confirmed: absent active dorsiflexion of the left ankle; significant weakness of the tibialis anterior, extensor hallucis longus, and peroneal muscles; decreased sensation along the lateral aspect of the lower leg and dorsum of the foot; positive foot slap during gait attempts. Musculoskeletal findings included: tightness of the left Achilles tendon, contributing to equinus deformity; swelling of the left foot with mild edema around the ankle; limited active and passive range of motion (AROM and PROM) of the left ankle, especially in dorsiflexion and eversion; severe pain and paresthesia, rated 5/10 on the Numerical Pain Rating Scale (NRS), localized to the lateral leg and dorsum of the foot. Functional assessment showed: complete dependence for mobility and nonambulatory status at admission; inability to bear weight, stand, or walk independently; significant impairment in activities of daily living (ADLs), particularly in bed‐to‐chair transfers, gait, and toileting. Electrophysiological studies were considered to further characterize the extent of nerve involvement. However, due to the acute wound condition, pain, and the need for early functional rehabilitation, formal electrophysiological testing was not performed before physiotherapy. The diagnosis of common peroneal nerve injury was therefore based on characteristic clinical findings, including motor weakness in ankle dorsiflexion and toe extension, sensory deficits over the dorsum of the foot, and functional gait impairment. Based on clinical and examination findings, a diagnosis of left CPNI secondary to a traumatic GSI was confirmed. Clinical progression and functional recovery were monitored over time to guide ongoing management. The study flow is shown in Figure 3.

## 4 Treatment

A structured, evidence‐based physiotherapy‐led rehabilitation program was initiated immediately upon the patient's admission. The goals were to: prevent secondary complications such as joint contractures and disuse atrophy, reduce pain and swelling, improve range of motion (ROM), muscle strength, and sensory feedback, and enhance functional mobility and independence. The physiotherapy‐led rehabilitation program lasted 3 months, consisting of 36 sessions. Treatment progress was regularly monitored and adjusted based on clinical improvements and functional goals. Treatment strategies included: pain and oedema management, range of motion and stretching exercises, muscle strengthening and electrical stimulation, sensory reeducation and motor retraining, balance and gait training, assistive device training, and functional task practice (Table 1).

**TABLE 1:** Structured physiotherapy and rehabilitation program.

Treatment strategies	Descriptions	Rationale/purpose
Pain and oedema management	– Cryotherapy (20 min) – Limb elevation	Reduce inflammation and pain [14, 15]
ROM exercises	– Passive and active‐assisted ROM of hip, knee, and ankle (10–20 reps per exercise)	Prevent joint stiffness, preserve joint mobility [14, 15]
Stretching program	– Static stretching: hip, knee, ankle muscles (15–30 s holds/3–5 reps)	Prevent contractures and maintain flexibility [14, 16]
Strengthening exercises	– Isometric exercise: Lower limb muscles (5 s hold/10 reps/1–3 sets) – Straight leg raises (10 s hold/10 reps) Progress to resisted exercise	Restore the strength of affected and unaffected muscles [14, 15]
Core stability	– Static back extension exercise (10 s hold/10 reps/2 sets)	Improve trunk control and posture [17]
Electrical stimulation	– EMS/TENS on affected limb (20 min)	Muscle reeducation and analgesia [14, 15, 16, 17]
Sensory reeducation	– Sensory integration with varied textures (cotton, rough cloth, sand, pebbles)	Enhance sensory input and motor output [14, 15]
Proprioception & motor retraining	– Ankle and foot mobilization – Weight‐shift and balance training	Improve joint awareness and neuromuscular control [18]
Orthotic support	– Prescription of Ankle–Foot Orthosis (AFO) – Worn during ambulation, removed at night	Maintain ankle alignment, assist gait [15, 16]
Gait training	– Initiated in parallel bars, progressing to walker – Gradual weight‐bearing (25% increments biweekly)	Restore independent ambulation safely [16]
Functional training	– Bed mobility – Sit‐to‐stand – Stair climbing – Ambulation on uneven surfaces	Reinforce daily task independence and ADL performance [14, 16]
Patient education	– Skin inspection, limb hygiene, orthosis care, counseling	Prevent secondary complications and promote self‐management [16]

## 5 Outcome and Follow‐Up

To comprehensively assess the patient's progress, a combination of structured and validated outcome measures was used. All outcomes were measured at baseline, after 3 months, and again after another 3 months for follow‐up evaluation. All outcome measures are listed below:

### 5.1 Self‐Structured Outcome Measure

**FIGURE 4:**
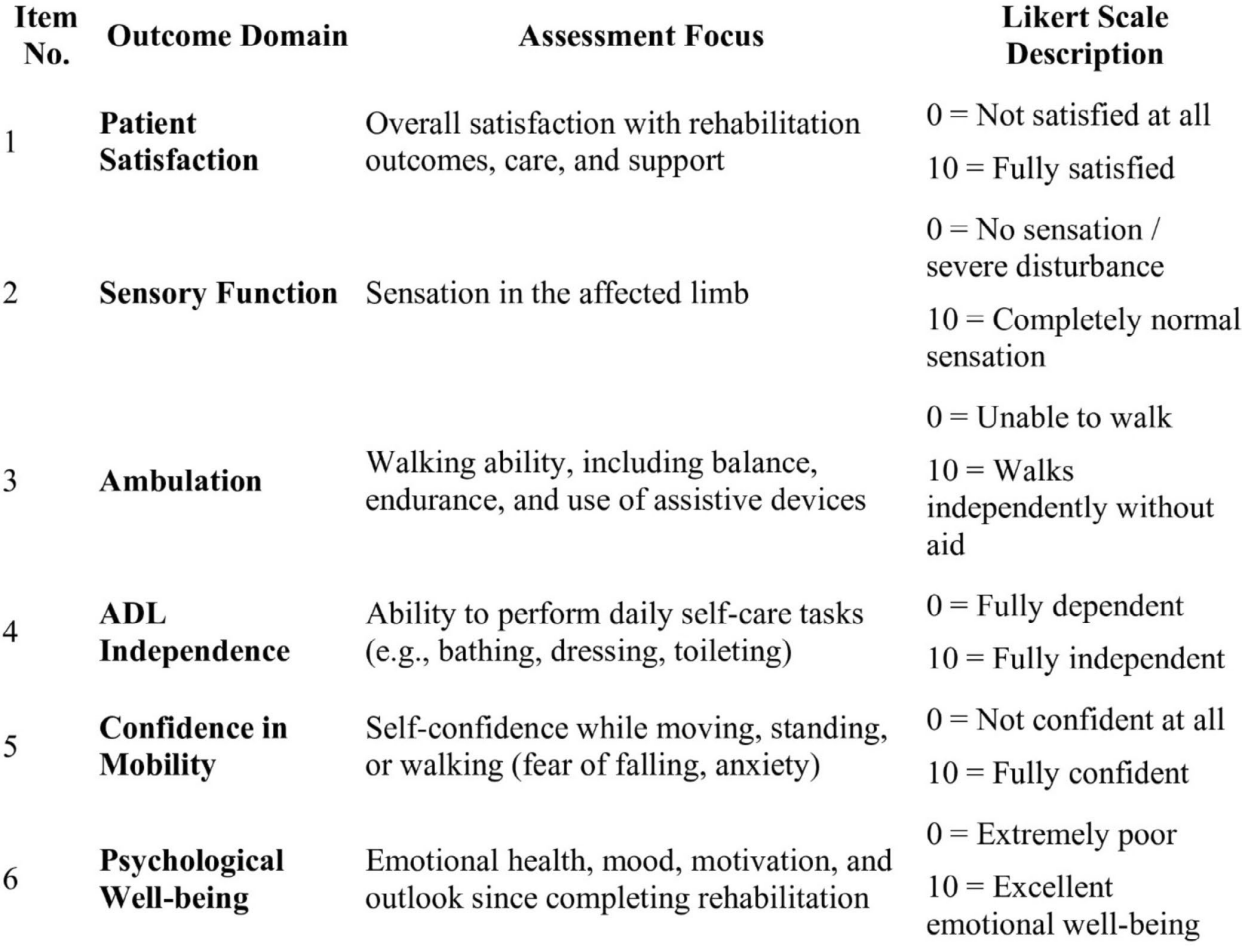
Self‐structured outcome measure.

A self‐structured outcome measure (SSOM) was created specifically for this study to assess post‐rehabilitation functional and psychosocial status in individuals with foot drop caused by GSI. The tool includes six domains: patient satisfaction, sensory function, ambulation, activities of daily living (ADL), confidence in mobility, and psychological well‐being. Each domain is scored on a 10‐point Likert scale (0 = worst, 10 = best), enabling measurement of patient‐perceived recovery (Figure 4).

### 5.2 Pain

Pain was assessed using validated outcome measures, the Numerical Pain Rating Scale (NRS). Patients are asked to choose the number that best represents their current pain level on a scale from 0 to 10, where (0) signifies no pain and (10) signifies the worst possible pain [19].

### 5.3 Muscle Strength

Muscle strength was assessed using a handheld dynamometer (HHD). The strength of the affected lower limb muscles, hamstrings, quadriceps, triceps surae, and tibialis anterior was objectively measured with this device, which provides a quantitative assessment of isometric muscle force. It is commonly used in clinical and rehabilitation settings for reliable evaluation of strength recovery [20].

### 5.4 Range of Motion

Range of motion (ROM) was measured using a standard universal goniometer (UG), a widely accepted and reliable tool in musculoskeletal and neurological rehabilitation. The goniometer was used to measure active joint ROM of the hip, knee, and ankle in degrees, following standard anatomical landmarks and procedures as outlined in previous research [21].

### 5.5 Walking Ability

The Six‐Min Walk Test (6MWT) is a standardized submaximal exercise test used to evaluate walking ability. It measures the total distance (in meters) an individual can walk on a flat, hard surface in 6 min, reflecting their integrated response [22].

### 5.6 Functional Ability

The Foot and Ankle Disability Index (FADI) is a validated patient‐reported outcome measure used to assess functional ability. It includes 26 items, four related to pain and 22 related to activities such as walking, stairs, standing, and daily tasks. Each item is scored on a 5‐point Likert scale (0 = unable to do; 4 = no difficulty), with a maximum score of 104 points. Higher scores indicate better functional ability and less disability [23].

### 5.7 Quality of Life

Quality of life was assessed using a validated questionnaire, the Short Form‐36 (SF‐36). This established tool measures health‐related quality of life across eight areas, including physical function, social limitations, pain, mental health, and vitality [24].

## 6 Results

**FIGURE 5:**
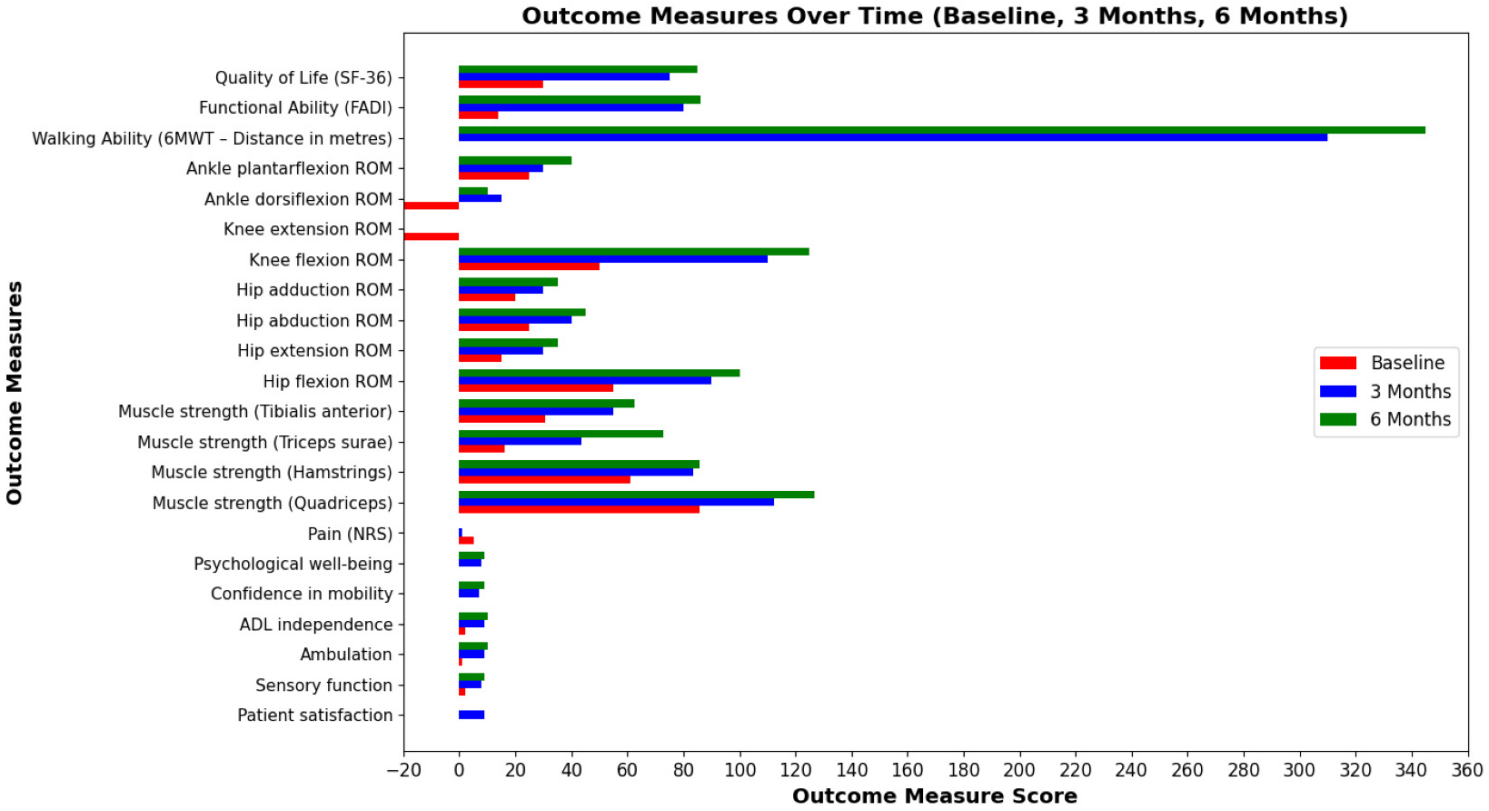
Outcome measures visualization.

Following 3 months (36 sessions) of structured physiotherapy‐led rehabilitation, the patient demonstrated clinically meaningful improvements across all outcome measures compared with baseline, with no adverse events reported. Post‐intervention assessment showed marked reductions in pain and significant improvements in muscle strength, joint range of motion, walking capacity, functional ability, and health‐related quality of life. At the 6‐month follow‐up, these gains were largely maintained, with further improvement observed in selected functional outcomes, a pattern commonly reported after the transition from supervised rehabilitation to continued activity. These findings suggest a beneficial role of adherence to the prescribed home‐based exercise program in sustaining and consolidating functional recovery. Overall functional status continued to improve, as evidenced by sustained independent ambulation, increased 6‐min walk distance, and stable or enhanced functional and quality‐of‐life scores, supporting the durability of the rehabilitation effects. The patient achieved independent mobility and was returned to ADLs without the need for assistive devices. Detailed outcome changes are presented in Tables 2 and 3 and Figure 5.

**TABLE 2:** Patient outcome measures.

Outcome measures	Baseline	At 3 months (posttest)	At 6 months (follow‐up)
Self‐structured outcome measure (SSOM)			
Patient satisfaction (/10)	—	9	—
Sensory function (/10)	2	8	9
Ambulation (/10)	1	9	10
ADL independence (/10)	2	9	10
Confidence in mobility (/10)	0	7	9
Psychological well‐being (/10)	0	8	9
Pain (NRS, /10)	5	1	0
Muscle Strength (HHD, lbs)			
Quadriceps	85.60	112.40	126.90
Hamstrings	61.20	83.50	85.90
Triceps surae	16.30	43.60	72.80
Tibialis anterior	30.60	55.10	62.40
Range of Motion (degrees)			
Hip flexion	55	90	100
Hip extension	15	30	35
Hip abduction	25	40	45
Hip adduction	20	30	35
Knee flexion	50	110	125
Knee extension	−20	0	0
Ankle dorsiflexion	−20	15	10
Ankle plantarflexion	25	30	40
Walking Ability (6MWT, meters)	0	310	345
Functional Ability (FADI, /104)	14	80	86
Quality of Life (SF‐36, /100)	30	75	85

Abbreviations: 6MWT, 6‐Min Walk Test; ADL, Activity of Daily Living; FADI, Foot–Ankle Disability Index; HHD, Handheld Dynamometer; NRS, Numeric Pain Rating Scale; SF‐36, Short Form Survery‐36.

**TABLE 3:** Change in outcome measures over time.

Outcome measures	Change from baseline to 3 months (T1)	Change from 3 months to 6 months (T2)	Change from baseline to 6 months (T3)
Self‐structured outcome measure (SSOM)			
Patient satisfaction (/10)	9	—	—
Sensory function (/10)	+6	+1	+7
Ambulation (/10)	+8	+1	+9
ADL independence (/10)	+7	+1	+8
Confidence in mobility (/10)	+7	+2	+9
Psychological well‐being (/10)	+8	+1	+9
Pain (NRS, /10)	−4	−1	−5
Muscle Strength (HHD, lbs)			
Quadriceps	+26.80	+14.50	+41.30
Hamstrings	+22.30	+2.40	+24.70
Triceps surae	+27.30	+29.20	+56.50
Tibialis anterior	+24.50	+7.30	+31.80
Range of Motion (ROM, degrees)			
Hip flexion	+35	+10	+45
Hip extension	+15	+5	+20
Hip abduction	+15	+5	+20
Hip adduction	+10	+5	+15
Knee flexion	+60	+15	+75
Knee extension	+20	0	+20
Ankle dorsiflexion	+35	−5	+30
Ankle plantarflexion	+5	+10	+15
Walking Ability (6MWT, meters)	+310	+35	+345
Functional Ability (FADI, /104)	+66	+6	+72
Quality of Life (SF‐36, /100)	+45	+10	+55

Abbreviations: 6MWT, 6‐Min Walk Test; ADL, Activity of Daily Living; FADI, Foot–Ankle Disability Index; HHD, Handheld Dynamometer; NRS, Numeric Pain Rating Scale; SF‐36, Short Form Survery‐36.

## 7 Discussion

GSIs to the lower extremity often cause complex neuromuscular trauma, with PNI, particularly to the CPN, being among the most common and disabling complications due to its superficial course around the fibular neck and limited soft tissue coverage. Damage to the CPN usually results in foot drop, impaired dorsiflexion, gait abnormalities, and a significant loss of functional independence. In this case, the patient presented with severe motor and sensory deficits secondary to a GSI‐induced CPN injury, leading to complete dependency in walking and activities of daily living (ADLs). Early and comprehensive rehabilitation was crucial for promoting neural recovery, preventing secondary complications, and maximizing functional outcomes [3, 4, 5, 6, 7]. The intervention plan was multifaceted, including neuromuscular reeducation, strength training, range of motion (ROM) exercises, balance retraining, orthotic management, and pain control strategies based on current neurorehabilitation guidelines and evidence [14, 15, 16, 17, 18]. Over 3 months, and maintained for the subsequent 3 months, the patient demonstrated significant clinical and functional improvements across all outcome measures. Outcome measures, such as the Numeric Rating Scale (NRS) for pain, improved from 5/10 to 1/10 at 3 months and were completely resolved by 6 months. Muscle strength notably increased, especially in the tibialis anterior (+31.8 lbs) and triceps surae (+56.5 lbs), emphasizing the benefits of progressive strengthening exercises and electrical stimulation in enhancing motor unit recruitment and neuroplasticity [14, 16, 17]. Joint range of motion (ROM) increased significantly, with ankle dorsiflexion improving from −20° at baseline to +10°, and knee flexion increasing by 75°, resulting in a more normalized gait pattern and a reduced risk of joint contractures. These improvements support research indicating that early mobilization and flexibility exercises help reduce joint stiffness and promote motor recovery after lower limb trauma [15, 16]. Functional walking, measured by the Six‐Min Walk Test (6MWT), increased from 0 m at baseline to 310 m at 3 months and 345 m at 6 months, indicating restored endurance, cardiovascular capacity, and lower limb coordination. The use of ankle–foot orthoses (AFOs) in the early stages further supported gait mechanics and safe ambulation in patients with foot drop [15, 16, 25]. The Foot and Ankle Disability Index (FADI) score improved substantially, from 14/104 at baseline to 80/104 at 3 months and 86/104 at 6 months, reflecting better function in activities such as standing, walking, stair climbing, and dynamic balance. These findings are backed by studies that show the FADI's sensitivity to interventions aimed at improving ankle and foot function in neurologically impaired populations [14, 15, 16, 17, 18].

Physiotherapy has consistently been identified as a key component in managing foot drop caused by CPN injury, particularly in traumatic contexts. The literature indicates that early, structured rehabilitation targeting joint mobility, progressive muscle strengthening, sensorimotor retraining, and gait reeducation can facilitate neuromuscular recovery and reduce long‐term disability. Interventions such as range‐of‐motion exercises and stretching help prevent joint stiffness and contracture, while progressive strengthening and neuromuscular electrical stimulation support motor unit recruitment during nerve recovery. Balance training, proprioceptive exercises, and task‐specific gait practice have been shown to enhance functional ambulation and reduce compensatory movement patterns. Temporary use of ankle–foot orthoses is commonly recommended to maintain ankle alignment and promote safe mobility during early rehabilitation [26]. The outcomes observed in the present case—improvements in muscle strength, walking capacity, functional independence, and quality of life—are consistent with the existing literature of physiotherapy‐led rehabilitation, reinforcing its role as an effective conservative management strategy for post‐traumatic foot drop.

It's also wonderful to see the patient's own progress, which was tracked using a self‐structured outcome measure (SSOM). This measure revealed significant improvements in sensory recovery (from 2 to 9/10), walking ability (from 1 to 10/10), independence in daily activities (from 2 to 10/10), and psychological confidence (from 0 to 9/10) over 6 months. The patient repeated outcome measures, and frequent evaluations were conducted to assess the effectiveness of the intervention. The patients were described as feeling extremely happy and satisfied after the session, which is truly inspiring.

Cryotherapy, elevation, TENS, and therapeutic exercises have been presented as highly effective methods for pain reduction. These highlight the importance of pain control in various ways, making rehabilitation easier to observe and respond to. Proprioceptive training and texture reeducation aid in developing sensitivity, which is vital for reducing the risk of falls and improving motor skills. The Short Form‐36 (SF‐36) score, which measures health‐related quality of life (HRQoL), improved significantly from 30/100 to 85/100 after treatment. This improvement reflects the significance of social, functional, and mobility aspects, as well as strength during recovery. In cases of PNI and post‐footdrop, early, well‐planned, and appropriate rehabilitation is essential. Research indicates that neuroplasticity, active patient participation, and activation of the visual system can induce significant changes, even in initially challenging conditions. This report demonstrates how targeted early exercise can assist patients and their clinicians in returning to near‐normal function, thereby avoiding long‐term disability. It also aligns with the previous findings for assessing functional‐specific rehabilitation outcomes. Sometimes, nerve repair or tendon transfer surgery may be necessary, but commencing treatment as early as possible remains important. Overall, this recovery journey shows how a tailored and physiotherapy‐focused rehab program can successfully treat GSI with foot drop, highlighting the importance of personalized care.

## 8 Strengths and Limitations

This case report demonstrates how effective a carefully designed and research‐supported physiotherapy‐based rehabilitation program can be in helping patients recover from post‐traumatic foot drop—a common complication of peripheral neuropathy (PNI). Reliable outcome measures such as the NRS, HHD, 6MWT, FADI, and SF‐36, as well as a patient‐centered, self‐reported instrument called the SSOM, were used to provide a continuous picture of the patient's physical and psychological recovery. The plan included orthotic support, functional training, and sensory reeducation over a 3‐month period, all of which contributed to the patient's lasting and meaningful improvement. Together, these aspects provide a strong foundation for this case report. However, the inherent limitations of a single case study make it difficult to generalize these results. Furthermore, the lack of posttreatment electrophysiological or imaging evaluations makes it difficult to obtain objective evidence of neurological recovery. In addition, the lack of validity of self‐reported outcome measures may limit the interpretation of results. In addition, the absence of comparative data with surgical or alternative treatment methods also limits medical decision‐making.

## 9 Conclusions

This case report demonstrates how conservative physiotherapy‐led rehabilitation can support functional recovery after foot drop and PNIs caused by gunshots. Mobility, muscular strength, and quality of life all improved significantly with early, tailored treatments and consistent follow‐up. These findings support physiotherapy‐led rehabilitation as a practical and effective approach, especially in settings with limited resources. Further research involving larger patient groups and comparative methodologies is necessary to strengthen the evidence. Future studies should also examine long‐term outcomes and cost‐effectiveness to inform therapeutic guidelines and improve management of PNIs following trauma.

## 10 Patient's Perspective

After getting shot in the leg and believing my life was finished, I felt as if everything had gone wrong forever. I'm in a lot of pain and not able to move my hurt leg at all. My family helped me with important things like getting around, getting dressed, and going to the bathroom. However, the emotional effects of sadness, the fear of being disabled for life, and losing self‐respect were even worse than not being able to move. At first, I didn't think I would be able to walk or get back on my own. When I got to this Rehabilitation Center, I wasn't feeling very hopeful and was rather worried. The physiotherapy team gave both organized help and mental support. Every session was hard on my body and sometimes made me feel uncomfortable, but they all helped me get my confidence, power, and ability to move again. The staff helped me understand my injuries, helped me set objectives, and provided the tools and knowledge I needed to actively participate in my recovery. As my healing went on, I began to notice real improvements. I was able to sit up unaided, move from bed to chair, and finally take steps independently. The things I had to do every day got easier over time. With consistent hard work, guided exercise treatments, and ongoing support from the physiotherapy team, I progressed from needing help with everything to being able to walk unaided. This change didn't happen all at once, but each step forward felt like reclaiming parts of my body, mind, and social life.

Today, I am capable of walking unaided, participating in social activities, and managing my personal care needs without help. Beyond the physical recovery, this journey has restored my self‐esteem and sense of purpose. I am deeply grateful to the rehabilitation professionals, whose holistic approach and unwavering support enabled me to return to an independent and dignified life.

## 11 Learning Points

- Gunshot injuries to the lower limb can lead to complex common peroneal nerve injuries, resulting in foot drop and severe functional disability.
- Timely initiation of a structured physiotherapy‐led rehabilitation program is critical to prevent contractures, restore mobility, and promote neuromuscular recovery.
- Evidence‐based physiotherapy incorporating pain management, range of motion exercises, progressive strengthening, sensory reeducation, and gait training can significantly improve outcomes without surgical intervention.
- Standardized outcome tools such as NRS, HHD, 6MWT, FADI, SF‐36, and SSOM allow for comprehensive tracking of motor, functional, and psychosocial recovery.
- This case underscores the feasibility and effectiveness of conservative, resource‐appropriate rehabilitation for traumatic nerve injuries in low‐ and middle‐income settings.

## Author Contributions

**Mahmuda Akter Akhi:** conceptualization, data curation, formal analysis, investigation, methodology, writing – original draft. **Kazi Md Azman Hossain:** investigation, methodology, project administration, visualization, writing – original draft, writing – review and editing. **Sabrina Tina:** methodology, validation, writing – review and editing. **Md. Abdul Alim:** project administration, resources, supervision, writing – review and editing.

## Funding

The authors have nothing to report.

## Ethics Statement

Ethical approval was obtained from the Institute of Physiotherapy Rehabilitation and Research of Bangladesh Physiotherapy Association (BPA) before the submission of this manuscript.

## Consent

Written informed consent was obtained directly from patient(s) for assessment, intervention, and to publish this report in accordance with the journal's patient consent policy.

## Conflicts of Interest

The authors declare no conflicts of interest.

## Data Availability

The authors have nothing to report.
